# AC-X: Characteristic Antinuclear Antibody Patterns of Two Anti-Mi-2 Autoantibody-Positive Dermatomyositis Patients—A Case Report

**DOI:** 10.3389/fimmu.2022.857030

**Published:** 2022-03-17

**Authors:** Ziyan Wu, Honglin Xu, Shulan Zhang

**Affiliations:** Department of Rheumatology and Clinical Immunology, Peking Union Medical College Hospital, Chinese Academy of Medical Sciences & Peking Union Medical College, National Clinical Research Center for Dermatologic and Immunologic Diseases (NCRC-DID), Ministry of Science and Technology; Key Laboratory of Rheumatology and Clinical Immunology, Ministry of Education, Beijing, China

**Keywords:** anti-Mi-2 autoantibody, dermatomyositis, antinuclear antibody patterns, AC-X, clinical routine

## Abstract

Here we reported two anti-Mi-2 autoantibody-positive dermatomyositis (DM) patients with a characteristic antinuclear antibody (ANA) immunofluorescence pattern. Autoantibodes were screened by indirect immunofluorescence (IIF) on HEp-2 cells (Euroimmun, Lübeck, Germany) and confirmed by line immunoblot (ANA Profile 3—Euroimmun, Germany). These two patients were positive for ANA (speckled, titer 1:320), followed by confirmation of positive anti-Mi-2α and anti-Mi-2β positive and negative for all other antibodies. We found a characteristic ANA pattern of the anti-Mi-2 antibody that differed from the AC-4 pattern, especially in the morphology of mitotic cells (metaphase, anaphase, and telophase). Thus, we would like to suggest reporting this characteristic antinuclear antibody pattern as a new AC type, as AC-X.

## Introduction

**Figure 1:**
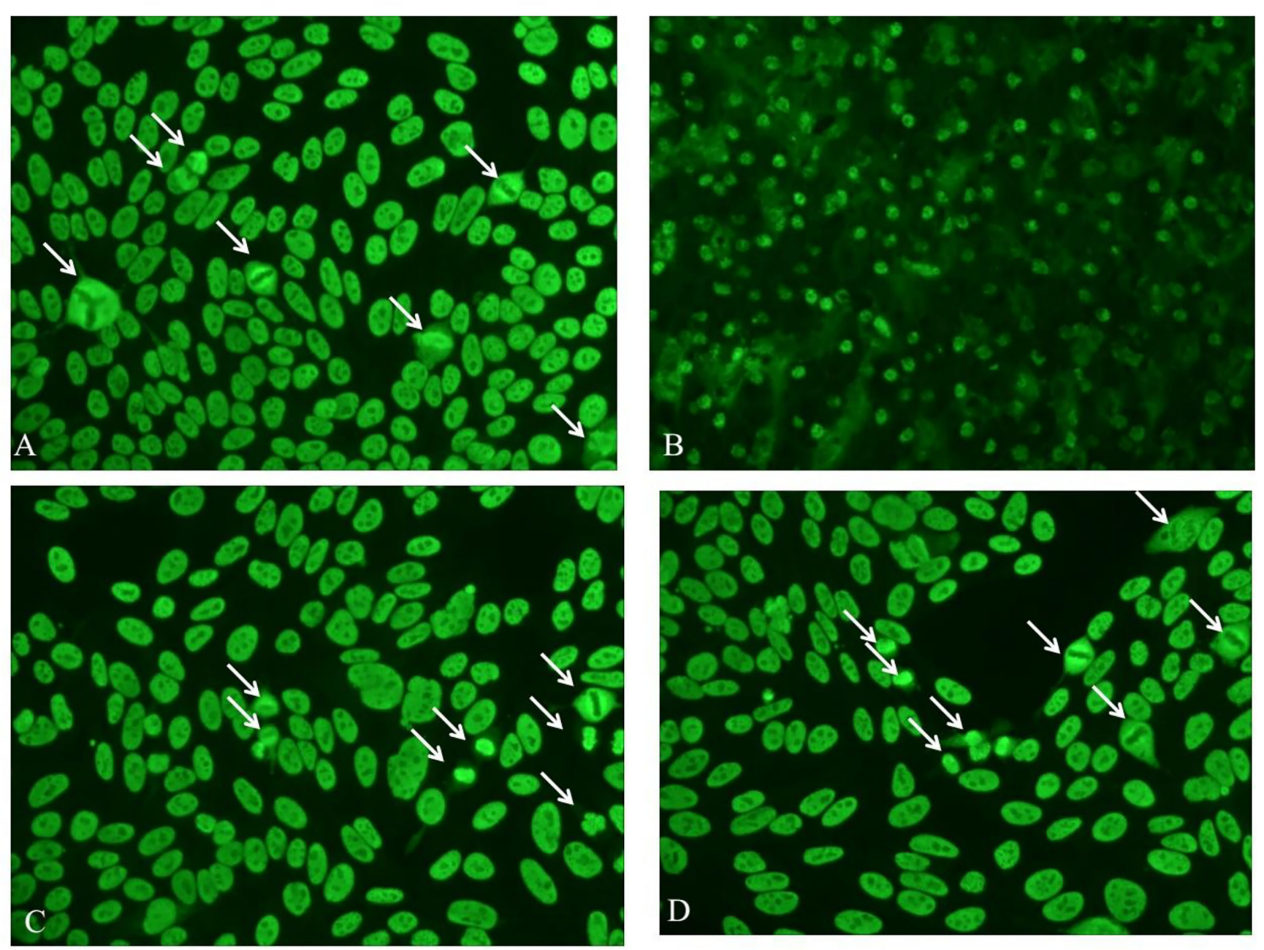
Immunofluorescence patterns of the 50-year-old DM patient by IIF, diluted 1:100, on the liver cryostat sections of mouse **(B)** and on Hep-2 cells **(A, C, D)**. White arrow-marked mitotic cells (metaphase, anaphase, and telophase) had the chromatin mass stained or not stained.

Here we reported two anti-Mi-2 autoantibody-positive dermatomyositis (DM) patients with a characteristic antinuclear antibody (ANA) immunofluorescence pattern. Two female patients, aged 50 and 73, were both diagnosed with DM with cutaneous lesions and muscle involvement. The clinical manifestation of the 50-year-old patient was bilateral eyelid erythema, periungual erythema, neck and back rash, and muscle weakness of the extremities, while the 73-year-old patient had developed rashes that first appeared on the right lateral thigh and spread on the face, buttocks, and extremities. In addition, the patient also suffered from muscle weakness in the extremities and pulmonary adenocarcinoma. Evaluated creatine kinase was found in both patients (738 U/L and 7211U/L, respectively). Autoantibodes were screened by indirect immunofluorescence (IIF) on HEp-2 cells (Euroimmun, Lübeck, Germany) and confirmed by line immunoblot (ANA Profile 3—Euroimmun, Germany). These two patients were positive for ANA (speckled, titer 1:320), followed by confirmation of positive anti-Mi-2α and anti-Mi-2β positive and negative for all other antibodies, such as anti-dsDNA, anti-SSA, anti-Ro52, anti-SSB, anti-RNP, anti-Sm, anti-Scl70, anti-Jo-1, anti-rRNP, anti-PCNA, anti-PM-Scl, anti-CENP, anti-M2, anti-OJ, anti-EJ, anti-PL-12, anti-PL-7, anti-SRP, anti-PM75, anti-PM100, anti-SAE1, anti-NXP2, anti-MDA5, and anti-TIFγ. The International Consensus on ANA Patterns (ICAP) had classified an anti-Mi-2-positive pattern into AC-4, also named nuclear fine speckled, which referred to fine tiny speckles throughout the nucleoplasm. Mitotic cells (metaphase, anaphase, and telophase) had the chromatin mass not stained (1). Interestingly, in our clinical routine, we found that it was a distinctive anti-Mi-2 antibody-positive ANA pattern that differed from the AC-4 pattern, particularly in the morphology of mitotic cells (metaphase, anaphase, and telophase). Anti-Mi-2 antibodies stained the interphase nucleoplasm outside of the nucleolus with a fine tiny speckled fluorescence pattern, but in contrast to the AC-4 pattern, the anti-Mi-2 antibody had a pleomorphic staining in mitotic cells (metaphase, anaphase, and telophase) (**Figure 1**). We found that mitotic cells (metaphase, anaphase, and telophase) had the chromatin mass stained or not stained (**Figure 1**), but not stained in AC-4. The stained condition of mitotic cells was one of the interpretation criteria for us to distinguish different ANA patterns. Thus, we would like to suggest reporting this characteristic ANA pattern as a new AC type, as AC-X. ANA detected by IIF underwent screening tests in most laboratories. The accurate interpretation of the ANA pattern was essential and urgent. Multiple nuclear dots and rim-like/membranes were the specific ANA patterns for the diagnosis of primary biliary cholangitis (2, 3). Although some positive ANA patterns were rare, it was vital for us to distinguish specific ANA patterns from common homogeneous or speckled staining ones.

The Mi-2 antigen was localized to the nucleus. It was a helicase of the nucleosome remodeling–deacetylase complex involved in transcription activation *via* two distinct enzymatic activities: histone deacetylase and ATP-dependent nucleosome remodeling, which implied that it would change in the process of cell mitosis simultaneously (4). It was a nuclear complex consisting of 8 protein components (240, 200, 150, 72, 65, 63, 50, and 34 kDa) and preferentially recognized 240 kDa as the major Mi-2 antigenic protein (5). Anti-Mi-2 autoantibodies immunoprecipitated two proteins, Mi-2α and Mi-2β, of 220 and 218 kDa, respectively. The anti-Mi-2 autoantibody was first identified in 1976 from a DM patient (named Mi-2) (6). It was a myositis-specific autoantibody, which was always associated with DM rather than polymyositis dermatomyositis (PM). Moreover, the frequency of the anti-Mi-2 autoantibody varied among different studies, with positive rates of 4%–59%. Anti-Mi-2 autoantibody positivity was associated with a good prognosis and a favorable response to corticosteroids in DM (7).

Moreover, the anti-Mi-2 autoantibody may present 3 months before DM-specific manifestations (8, 9). It was not only a specific biomarker for the diagnosis of DM, a reminder for the predictive and prognosis of DM (10). Therefore, it was urgent to reconsider the classification of suspicious anti-Mi-2 autoantibody-positive morphological performance by IIF on Hep-2 cells for more efficient ANA reflex testing. It would be classified into a new AC type as AC-X. Specific autoantibodies tested by ELISA, immunoblotting, or chemiluminescence were also strongly recommended.

## Data Availability Statement

The original contributions presented in the study are included in the article/supplementary material. Further inquiries can be directed to the corresponding author.

## Ethics Statement

The studies involving human participants were reviewed and approved by JS-2156. The patients/participants provided their written informed consent to participate in this study. Written informed consent was obtained from the individual(s) for the publication of any potentially identifiable images or data included in this article.

## Author Contributions

ZW and HX wrote the manuscript. SZ supervised the whole process. All authors contributed to the article and approved the submitted version.

## Funding

This research was supported by grants from the National Natural Science Foundation of China Grants (81801631, 81771661).

## Conflict of Interest

The authors declare that the research was conducted in the absence of any commercial or financial relationships that could be construed as a potential conflict of interest.

## Publisher’s Note

All claims expressed in this article are solely those of the authors and do not necessarily represent those of their affiliated organizations, or those of the publisher, the editors and the reviewers. Any product that may be evaluated in this article, or claim that may be made by its manufacturer, is not guaranteed or endorsed by the publisher.
